# Three-Dimensional Printing Assisted Laparoscopic Partial Nephrectomy vs. Conventional Nephrectomy in Patients With Complex Renal Tumor: A Systematic Review and Meta-Analysis

**DOI:** 10.3389/fonc.2020.551985

**Published:** 2020-10-22

**Authors:** Yingcheng Jiang, Huimin Zeng, Zewu Zhu, Jinbo Chen, Hequn Chen

**Affiliations:** Xiangya Hospital, Central South University, Changsha, China

**Keywords:** three-dimensional printing, laparoscopic partial nephrectomy, complex renal tumor, eGFR, meta-analysis

## Abstract

**Objective:** The purpose of this meta-analysis was to systematically assess the influence of three-dimensional (3D) printing technology in laparoscopic partial nephrectomy (LPN) of complex renal tumors.

**Methods:** A systematic literature review was performed in June 2020 using the Web of Science, PubMed, Embase, the Cochrane library, the China National Knowledge Infrastructure (CNKI), and the Wanfang Databases to identify relevant studies. The data relative to operation time, warm ischemic time, intraoperative blood loss, positive surgical margin, reduction in estimated glomerular filtration rate (eGFR), and complications (including artery embolization, hematoma, urinary fistula, transfusion, hematuria, intraoperative bleeding, and fever) were extracted. Two reviewers independently assessed the quality of all included studies, and the eligible studies were included and analyzed using the Stata 12.1 software. A subgroup analysis was performed stratifying patients according to the complexity of the tumor and surgery type or to the nephrometry score.

**Results:** One randomized controlled trial (RCT), two prospective controlled studies (PCS), and seven retrospective comparative studies (RCS) were analyzed, involving a total of 647 patients. Our meta-analysis showed that there were significant differences in operation time, warm ischemic time, intraoperative blood loss, reduction in eGFR, and complications between the LPN with 3D-preoperative assessment (LPN-3DPA) vs. LPN with conventional 2D preoperative assessment (LPN-C2DPA) groups. Positive surgical margin did not differ significantly.

**Conclusion:** The LPN-3DPA group showed shorter operation time and warm ischemic time, as well as less intraoperative blood loss, reduction in eGFR, fewer complications for patients with complex renal tumor. Therefore, LPN assisted by three-dimensional printing technology should be a preferable treatment of complex renal tumor when compared with conventional LPN. However, further large-scale RCTs are needed in the future to confirm these findings.

## Introduction

With the advancement and widespread usage of image technology, low-stage and small renal tumors are being detected more often in recent years, which has partly contributed to the dramatically increased incidence of renal tumors (1). Currently, there are three methods for partial nephrectomy: open surgery, laparoscopy, and robot-assisted laparoscopy. Since partial nephrectomy (PN) achieves equivalent oncological prognosis and lower incidence of adverse outcomes in comparison with radical nephrectomy (2), PN has gradually been recognized as a standard treatment for patients with clinically localized renal cell carcinoma with tumor size <4 cm and stage T1a based on the renal cell carcinoma guidelines (3). In recent years, with the advancement of technology, application of a robot-assisted surgery system has become a popular trend in the field of urology surgery. The robot surgery system has a highly flexible robotic arm and manipulators as well as a high-definition three-dimensional (3D) operating system; it has the characteristics of accurate operation, fine anatomy, and clear vision; and with it surgeons can perform the most precise operations.

Previously, surgeons defined pathologies and applied a surgical approach using a conventional two-dimensional (2D) monitor projecting X-ray, computer tomography, and magnetic resonance image scans. However, to define more complex lesions, including invisible feeding arteries and hilar or endophytic masses, conventional 2D preoperative assessment neither provides a sense of perspective nor does it facilitate these procedures (4). In recent decades, with the more widespread application of 3D printing technology in the medical field, doctors can obtain physical anatomical models based on patients' imaging data for preoperative assessment. In addition, a 3D-printed model can be used to study complex cases, to simulate and practice operations, to teach students, and to educate patients (5).

It is unclear whether or not patients with complex renal tumors benefit from a 3D-preoperative assessment. Recently, several studies have directly compared surgical outcomes and oncological outcomes of laparoscopic partial nephrectomy (LPN) with 3D-preoperative assessment (LPN-3DPA) vs. LPN with conventional 2D preoperative assessment (LPN-C2DPA) for complex renal tumors, but to date conclusions remain inconsistent. Therefore, it is necessary to conduct a systematic review and meta-analysis of evidence to evaluate the efficacy and safety of LPN-3DPA and in order to draw a more definitive and meaningful conclusion relative to its application.

## Methods and Materials

### Study Design

Article selection proceeded according to the search strategy based on the Preferred Reporting Items for Systematic Reviews and Meta-Analyses (PRISMA) guidelines (6).

### Participants, Interventions, and Comparator

Patients aged >16 years with complex renal tumor confirmed by pathology were enrolled in this study.

### Interventions

#### Treatment Group

Laparoscopic partial nephrectomy with 3D-preoperative assessment (LPN-3DPA).

#### Control Group

Laparoscopic partial nephrectomy with conventional 2D-preoperative assessment (LPN-C2DPA).

### Outcomes

The following parameters were analyzed to determine the advantages of 3D-preoperative assessment: (1) perioperative parameters, including operation time, warm ischemic time, intraoperative blood loss; (2) clinical outcomes, including positive margins and reduction in estimated glomerular filtration rate (eGFR); and (3) complications.

### Search Strategy

Two authors independently systematically searched the electronic literature databases. The search was performed in June 2020, and the Web of Science, PubMed, Embase, the Cochrane library, the China National Knowledge Infrastructure (CNKI), and the Wanfang Database were searched to identify relevant studies. No regional, language restrictions were set. The following were the MeSH term and text words used: “laparoscopic partial nephrectomy,” “3D,” “3 Dimensional,” “three dimensions,” “three-dimension,” “three dimensional,” “printing” were applied in search engines. In addition, the cited references of all selected articles were also further assessed for potentially relevant papers.

### Eligibility Criteria

The study was included in this meta-analysis if (1) it was a randomized controlled trial (RCT) or a non-RCT; (2) it reported the 3D printing-assisted LPN vs. conventional LPN for renal tumors; (3) 3D printing technology was only used for preoperative preparation; (4) studies recorded at least one of the following outcomes for LPN groups with both 3D printing preoperative assessment and conventional 2D preoperative assessment: operation time, warm ischemic time, intraoperative blood loss, reduction in eGFR, positive surgical margin, or complications. Exclusion criteria were as follows: (1) case reports, letters, conference abstracts, review articles, or meta-analysis; (2) duplicated publications from the same author or organization; (3) studies lacking sufficient data for extraction; (4) lack of the nephrometry score or evidence to assess the complexity of the tumor.

### Selection of Studies

The selection of included studies was conducted independently by two authors based on the PRISMA flow diagram, and the search results were imported into the software Endnote X9.3.3 (Thomson Corporation, USA). Firstly, we screened the titles and abstracts, and excluded the duplicated and apparently irrelevant references. Then, the full-text of the remaining potential studies were downloaded and reviewed to exclude those that did not meet our inclusion criteria. Finally, all disagreements were resolved by a third independent author until a consensus was reached.

### Data Extraction

Data were extracted and summarized from the included studies by two authors independently, and the consistency between them was checked by the third author. The extracted items were the following: (1) the general study information, including the first author, year of publication, study type, patients enrolled, age, sex, body mass index, tumor size, RENAL score, and PADUA score; (2) perioperative parameters, including operation time, warm ischemic time, intraoperative blood loss; (3) clinical outcomes, including positive margins and reduction in eGFR; (4) complications, including artery embolization, hematoma, urinary leakage, transfusion, hematuria, intraoperative bleeding, and fever. The continuous data were extracted as mean, SD (standard deviation), and the sample size. The dichotomous data were recorded as the number of events and the number of non-events.

### Assessment of Study Quality

The methodological quality of the studies was assessed using the Risk of Bias Tool recommended by the Cochrane Collaboration for RCT (7) and the Newcastle-Ottawa Scale (NOS) for non-RCT (8).

### Statistical Analyses

Data of the included studies were collected and STATA 12.1 (StataCorp LP, College Station, TX, USA) was applied for the meta-analysis. Statistical heterogeneity was assessed using the *I*^2^ statistic. The fixed-effects model was applied if no significant heterogeneity was detected or the statistical heterogeneity was low (*I*^2^ ≤ 50%). Otherwise, a random-effects model was used (*I*^2^ > 50%). For heterogeneity data, subgroup analysis was performed to identify the possible sources of heterogeneity. Subgroup analysis was performed by stratifying by complexity of the tumor, surgery type, or nephrometry score. Sensitivity analysis was conducted by consecutively omitting one single study to evaluate the reliability of the pooled results. The standard mean difference (SMD) was used for continuous outcomes and the odds ratio (OR) was used for dichotomous outcomes, both with 95% confidence interval (CI). For studies presenting continuous data as means and range, standard deviations (SDs) were analyzed using the technique described by Hozo et al. (9). Funnel plots and the Begg's test were applied to assess publication bias, and a *p* < 0.1 was defined as significant publication bias (10). The trim-and-fill computation was used to estimate the effect of publication bias on the interpretation of the results.

## Results

### Included Studies

**Figure 1:**
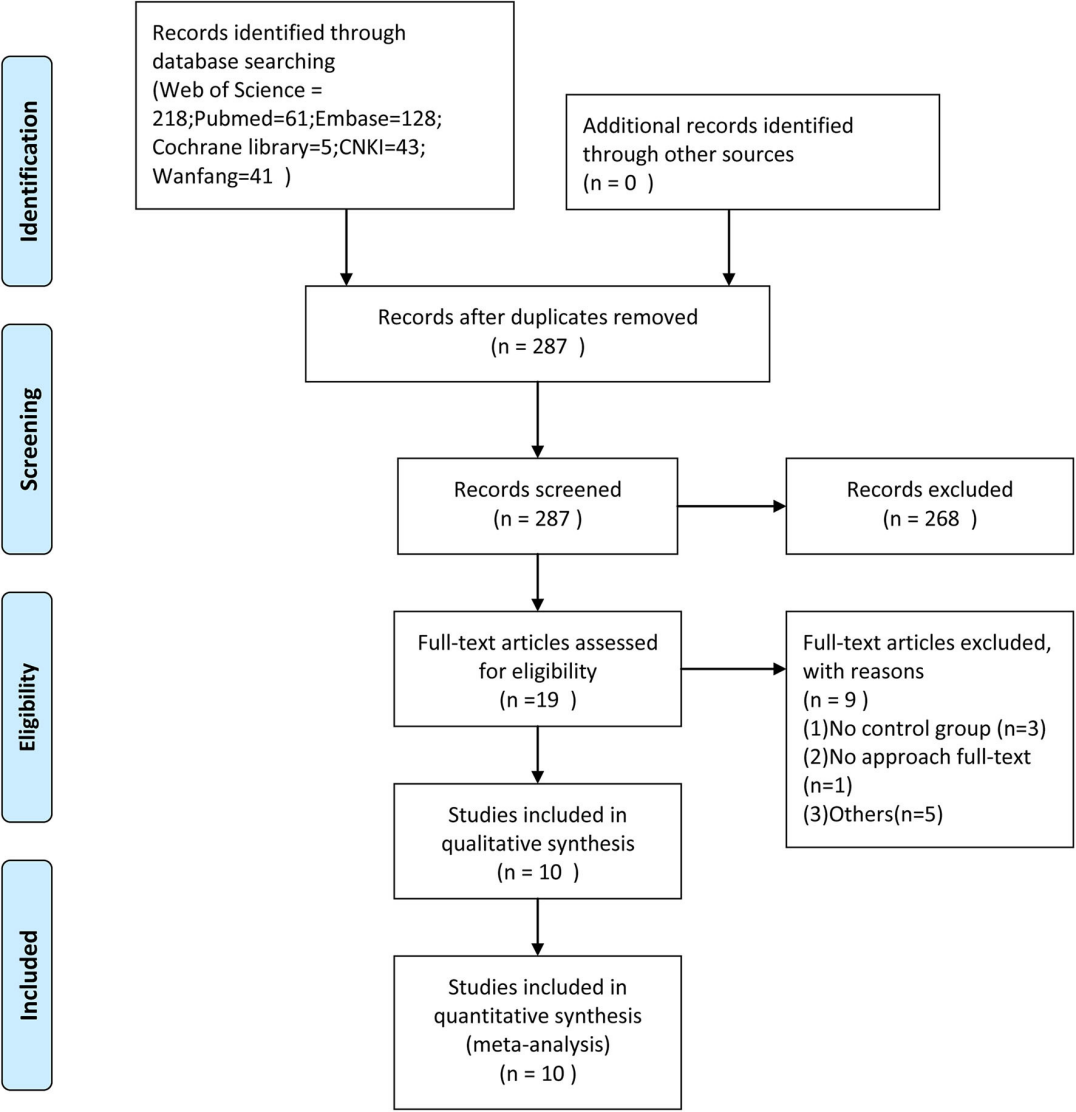
Flowchart of study selection.

A total of 496 candidate publications were identified through the Web of Science (*n* = 218), PubMed (*n* = 61), Embase (*n* = 128), Cochrane library (*n* = 5), CNKI (*n* = 43), and the Wanfang (*n* = 41) databases. After excluding the duplicate studies, 287 articles were screened for relevance on the basis of the title and abstract. Of the 19 articles that were deemed to meet the inclusion criteria based on the content of titles and abstracts, 9 were excluded for reasons of “no control group in the papers” and for other reasons (details are shown in Figure 1). The remaining 10 studies were included in the meta-analysis.

### Characteristics and Qualifications of Included Studies

The basic characteristics of all 10 included studies (11–20) were summarized and are shown in Table 1. All studies were published between 2017 and 2020. Trial sample sizes ranged from 20 to 127 patients, for a total of 647 patients with renal tumors that were enrolled in our meta-analysis: 322 in the experimental group and 325 in the control group. Risk of bias assessment of RCT is presented in Table 2. The Newcastle–Ottawa Scale was used to assess the risk of bias of the retrospective comparative studies (RCS) and prospective controlled studies (PCS), and the total scores of 5–9 indicated that the study was a low risk of bias (Table 3).

**Table 1:** The characteristics of the included studies.

**Study**	**Year**	**Study type**	**Patients enrolled**	**Age (years)**	**Gender**	**BMI (kg/m^**2**^)**	**Tumor size (cm)**	**RENAL score**			**PADUA score**
			**T/C**	**mean ± SD**	**male/female**	**mean ± SD**	**mean ± SD**	**Range**	**T/C**	**mean ± SD**	**mean ± SD**
Fan et al.	2019	RCS	69/58	T:48 ± 11.9 C:50 ± 11.4	T:38/31 C:26/32	T:23.7 ± 3 C:23.2 ± 4.4	T:4 ± 2.6 C:3.9 ± 1.4	4–7 8–12	33/39 36/19	NA	NA
Francesco et al.	2018	PCS	21/31	T:60.8 ± 12.3 C:59.5 ± 10.6	T:15/6 C:23/8	T:24 ± 1.5 C:25 ± 1.1	T:5.08 ± 1.61 C:5.09 ± 1.51	NA	NA	NA	T:11 ± 0.74 C:10.5 ± 0.74
Francesco et al.	2019	PCS	48/43	T:62 ± 15 C:58 ± 9.8	T:35/13 C:33/10	T:24.1 ± 3.7 C:25.9 ± 3.8	T:4.86 ± 1.87 C:4.46 ± 1.31	NA	NA	NA	T:11 ± 1.48 C:10 ± 0.74
Hu et al.	2018	RCS	42/46	T:50 ± 12.75 C:50.5 ± 14	T:25/17 C:26/20	T:24.74 ± 4.5 C:24.6 ± 3.9	T:3.8 ± 2.0 C:3.6 ± 1.6	4–10	42/46	NA	NA
Liu et al.	2019	RCS	12/14	T:53 ± 17.9 C:54 ± 11.3	T:7/5 C:8/6	T:22.89 ± 1.9 C:23.1 ± 1.5	T:3.52 ± 0.97 C:3.96 ± 1.04	NA	NA	T:6.5 ± 1.9 C:6.3 ± 1.5	NA
Sun et al.	2019	RCT	10/10	T:50.3 ± 19.2 C:58.1 ± 10.3	T:7/3 C:9/1	T:21.8 ± 1.7 C:22.7 ± 1.6	T:3.2 ± 1 C:3 ± 1	NA	NA	T:7.20 ± 1.55 C:7.00 ± 1.41	NA
Wang et al.	2019	RCS	21/28	T:56.25 ± 5.75 C:60 ± 6	T:15/6 C:17/11	T:23.25 ± 2.25 C:24 ± 1.75	T:3.2 ± 0.55 C:3.3 ± 0.475	8–12	21/28	T:10 ± 1 C:10 ± 0.75	NA
Wang et al.	2017	RCS	49/45	T:53.9 ± 8.6 C:56.8 ± 7.6	T:29/20 C:22/23	T:22.1 ± 1.6 C:22.0 ± 1.8	T:3.2 ± 1.5 C:3.4 ± 1.6	4–7 8–12	27/27 22/18	T:7.3 ± 1.7 C:6.9 ± 1.8	NA
Wu et al.	2020	RCS	20/20	T:58.95 ± 11.69 C:54.15 ± 11.90	T:15/5 C:14/6	T:25.12 ± 2.75 C:24.98 ± 2.61	T:5.05 ± 0.63 C:4.95 ± 0.67	4–6 7–9 10–12	3/3 14/15 3/2	NA	NA
Wu et al.	2020	RCS	30/30	T:57.6 ± 11.7 C:56.4 ± 9.8	T:22/8 C:21/9	T:25.2 ± 2.8 C:24.9 ± 2.5	T:4.0 C:3.75	4–6 7–9 10–12	11/12 17/15 2/3	NA	NA

*T, 3D group; C, Conventional group; SD, standard deviation; RCS, retrospective comparative studies; RCT, randomized controlled trial; PCS, prospective comparative studies; BMI, body mass index; NA, not available*.

**Table 2:** Risk of bias assessment of the randomized controlled trial.

**Study**	**Random sequence generation**	**Allocation concealment**	**Blinding of participants and personnel**	**Blinding of outcome assessment**	**Incomplete outcome data**	**Selective reporting**	**Other bias**
Sun et al.	Low risk	Unclear risk	High risk	Unclear risk	Low risk	Low risk	Low risk

**Table 3:** Risk of bias assessment of the retrospective comparative studies and prospective controlled studies.

**Study**	**Selection**				**Comparability**	**Outcome**			
	**Exposed cohort**	**Non-exposed cohort**	**Ascertainment of exposure**	**Outcome of interest**		**Assessment of outcome**	**Length of follow-up**	**Adequacy of follow-up**	**Total score**
Fan et al.	*	*	*	*	*	*	*	*	8
Francesco et al.	*	*	*	*	**	*	*	*	9
Francesco et al.	*	*	*	*	**	*	*	*	9
Hu et al.	*	*	*	*	*	*	*	*	8
Liu et al.	*	*	*	*	*	*	*	*	8
Wang et al.	*	*	*	*	*	*	*	*	8
Wang et al.	*	*	*	*	*	*	*	*	8
Wu et al.	*	*	*	*	*	*	*	*	8
Wu et al.	*	*	*	*	*	*	*	*	8

Risk of bias was assessed with use of the Newcastle–Ottawa Scale. “*” means a score of 1; “**” means a score of 2; the total score of this scale is 9. A higher overall score corresponds to a lower risk of bias; a total score of 5 or less indicates a high risk of bias.

### Operation Time

**Figure 2:**
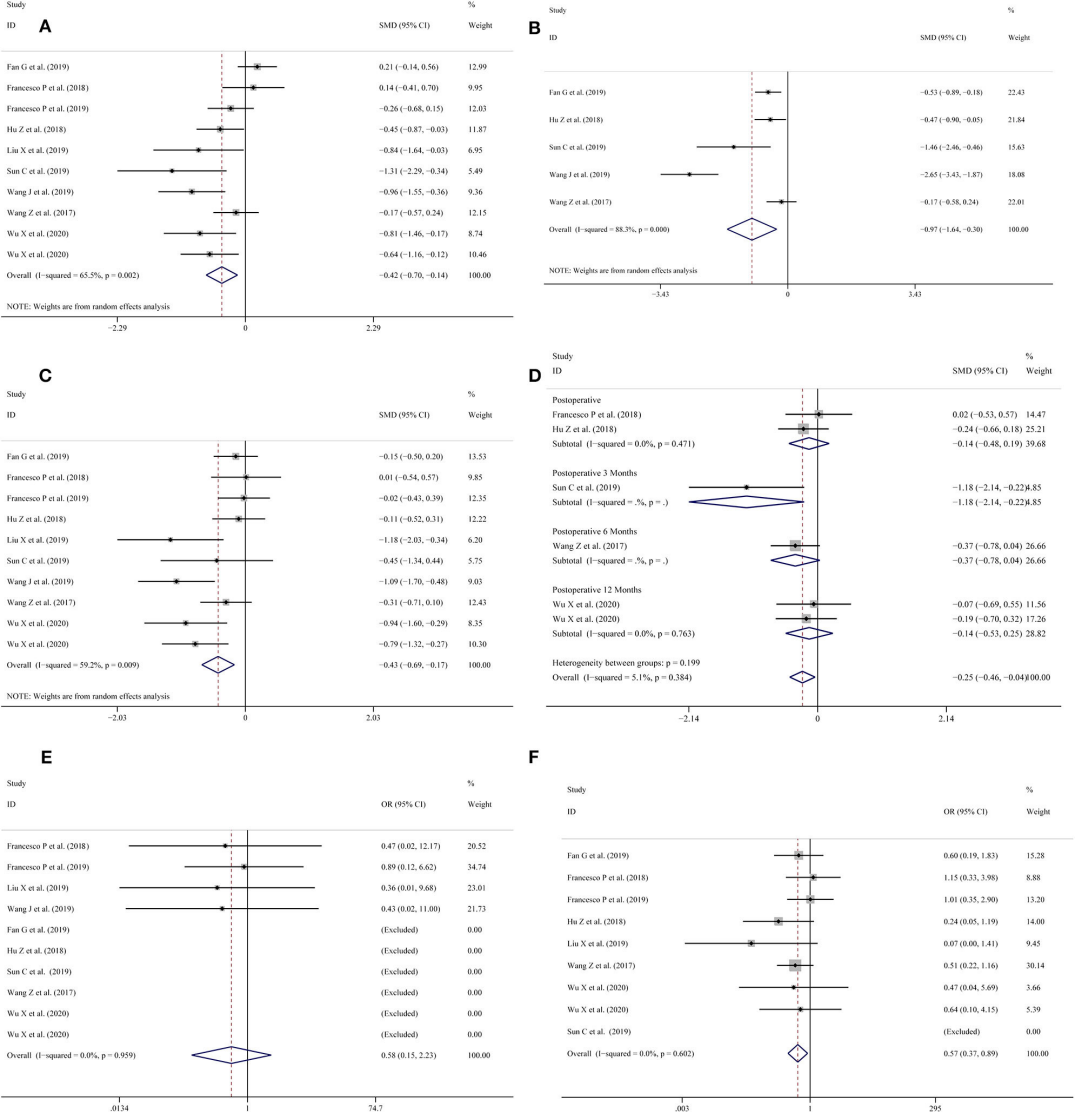
Forest plots for **(A)** operation time; **(B)** warm ischemia time; **(C)** intraoperative blood loss; **(D)** reduction in eGFR; **(E)** positive surgical margin; **(F)** complications.

All included studies (11–20) reported operation time. The pooled results of the meta-analysis showed that the 3D-preoperative assessment shortened the operation time (SMD = −0.42; 95% CI = −0.70 to −0.14, *I*^2^ = 65.5%, *P* = 0.002; Figure 2A) compared to conventional 2D-preoperative assessment surgeries.

### Warm Ischemia Time

Five studies (11, 13–16) provided data on the warm ischemia time. The pooled meta-analysis results showed that the use of 3D-preoperative assessment had a shortened warm ischemia time (SMD = −0.97; 95% CI = −1.64 to −0.30, *I*^2^ = 88.3%, *P* = 0.000; Figure 2B) in comparison with the traditional 2D-preoperative assessment surgeries.

### Intraoperative Blood Loss

Intraoperative blood loss was measured in 10 studies (11–13, 15–20); the meta-analysis showed that the use of 3D-preoperative assessment significantly reduced blood loss during surgery (SMD = −0.43; 95% CI = −0.69 to −0.17, I^2^ = 59.2%, P = 0.009; Figure 2C).

### Reduction in eGFR

Six studies (13, 15, 16, 18–20) collected records of the patients' eGFR reduction. Two (16, 18) studies recorded results post-operatively and one (13) study reported results 3 months post-operatively. The meta-analysis showed that there was no statistically significant difference between the LPN-3DPA and LPN-C2DPA groups post-operatively (OR = −0.14; 95% CI = −0.48 to 0.19, *I*^2^ = 0%, *P* = 0.471; Figure 2D). Another study (15) reported a reduction in eGFR at 6 months post-operatively and two studies (19, 20) reported similar reductions 12 months post-operatively, respectively. The pooled result indicated that there was no statistically significant difference between these two groups at 12-months post-operatively (OR = −0.14; 95% CI = −0.53 to 0.25, *I*^2^ = 0%, *P* = 0.763; Figure 2D). Overall, a significant difference was found between the two groups (OR = −0.25; 95% CI = −0.46 to −0.04, *I*^2^ = 5.1%, *P* = 0.384; Figure 2D).

### Positive Surgical Margin

All studies (11, 13–16, 18–22) reported a positive surgical margin. Overall, no significant differences were found between the two groups (OR = 0.58; 95% CI = 0.15–2.23, *I*^2^ = 0.0%, *P* = 0.959; Figure 2E).

### Complications

**Figure 3:**
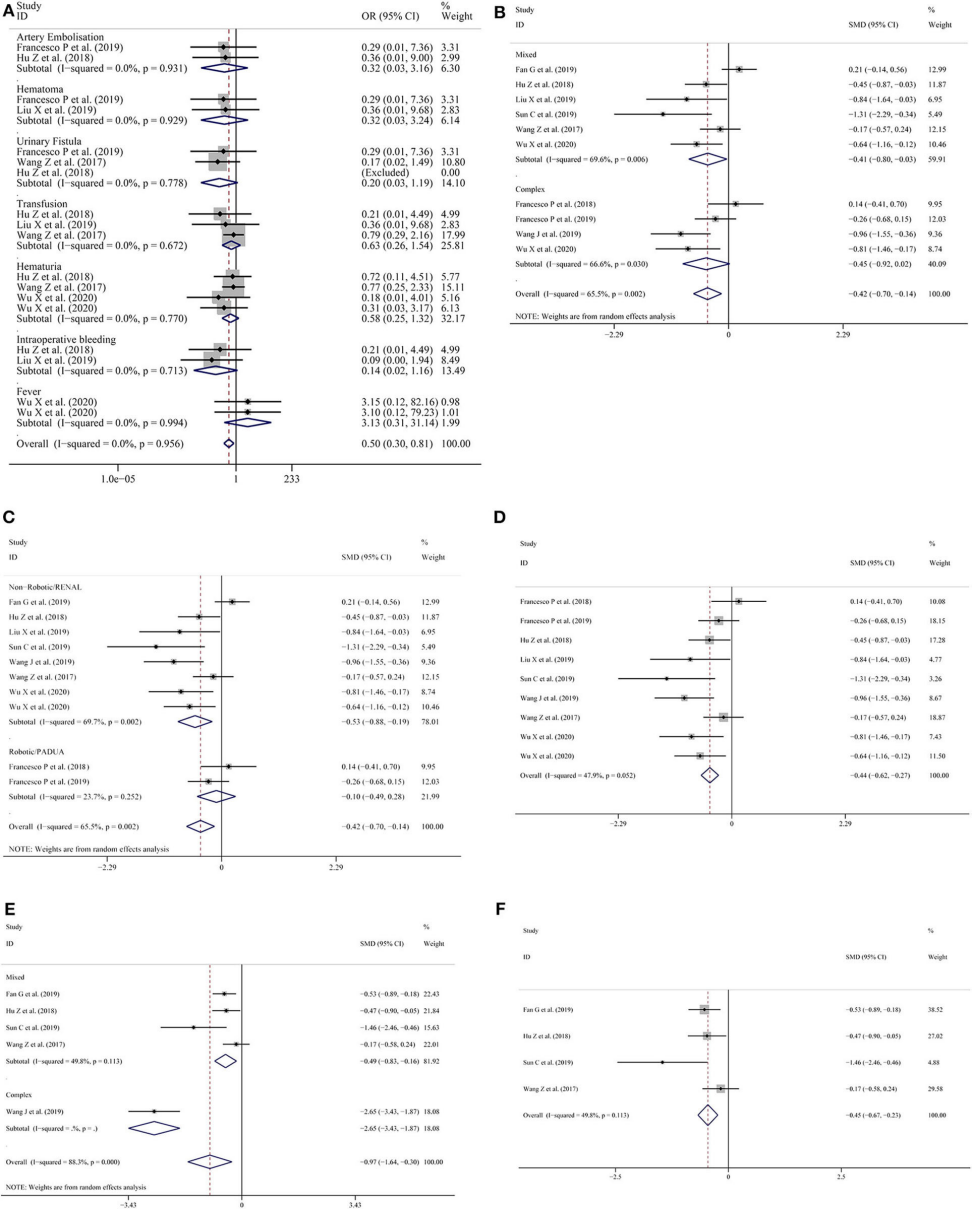
**(A)** Forest plots for single complications; **(B)** subgroup analysis performed by complexity of the tumor for operation time; **(C)** subgroup analysis performed by type of surgery or nephrometry score for operation time; **(D)** operation time excluding the study by Fan et al.; **(E)** subgroup analysis performed by complexity of the tumor for warm ischemia time; **(F)** warm ischemia time excluding the study by Wang et al.

Nine studies (11, 13–15, 18–22) provided data on complications. The pooled results showed that the LPN-3DPA group had a lower incidence of overall complications than the LPN-C2DPA group (OR = 0.57; 95% CI = 0.37–0.89, *I*^2^ = 0.0%, *P* = 0.602; Figure 2F). Reported complications included artery embolization, hematoma, urinary fistula, transfusion, hematuria, intraoperative bleeding, and fever. Conversely, to avoid complications from being overly reported, we counted the incidence of the above complications, respectively. The pooled results for each single complication indicated that there was a trend that the LPN-3DPA group was associated with a lower incidence of each complication, although it failed to reach a significantly statistical difference (Figure 3A).

### Subgroup Analysis

In view of the high level of heterogeneity, we conducted a subgroup analysis in which the studies were categorized into subgroups according to the complexity of the tumor and surgery type or the nephrometry score.

In terms of operative time during subgroup analysis, high heterogeneity was found in the mixed tumor group and complex tumor group (Figure 3B). But when the subgroup analysis was stratified by type of surgery or nephrometry score, the heterogeneity was low (SMD = −0.10; 95% CI = −0.70 to −0.14, *I*^2^ = 23.7%, *P* = 0.252; Figure 3C) in the studies with robotic surgery or PADUA nephrometry score group, but the heterogeneity was high (SMD = −0.53; 95% CI = −0.88 to −0.19, *I*^2^ = 69.7%, *P* = 0.002; Figure 3C) in studies with non-robotic surgery or RENAL nephrometry score. After the study by Fan et al. (11) was excluded, the overall heterogeneity declined dramatically (SMD = −0.44; 95% CI = −0.62 to −0.27, *I*^2^ = 47.9%, *P* = 0.052; Figure 3D).

In terms of the warm ischemic time for the subgroup analysis, because studies with robotic surgery or PADUA nephrometry score do not record the warm ischemic time, we only conducted subgroup analysis by complexity of the tumor. The results showed that lower heterogeneity was found in the mixed tumor group (SMD = −0.49; 95% CI = −0.83 to −0.16, *I*^2^ = 49.8%, *P* = 0.113; Figure 3E) compared with the overall groups (SMD = −0.97; 95% CI = −1.64 to −0.30, *I*^2^ = 88.3%, *P* = 0.000; Figure 3E). To reduce the pooled result heterogeneity, we consecutively omitted included studies one by one. After Wang et al.'s study (14) was excluded, the heterogeneity relative to the warm ischemic time declined significantly (SMD = −0.45; 95% CI = −0.67 to −0.23, *I*^2^ = 49.8%, *P* = 0.113; Figure 3F).

**Figure 4:**
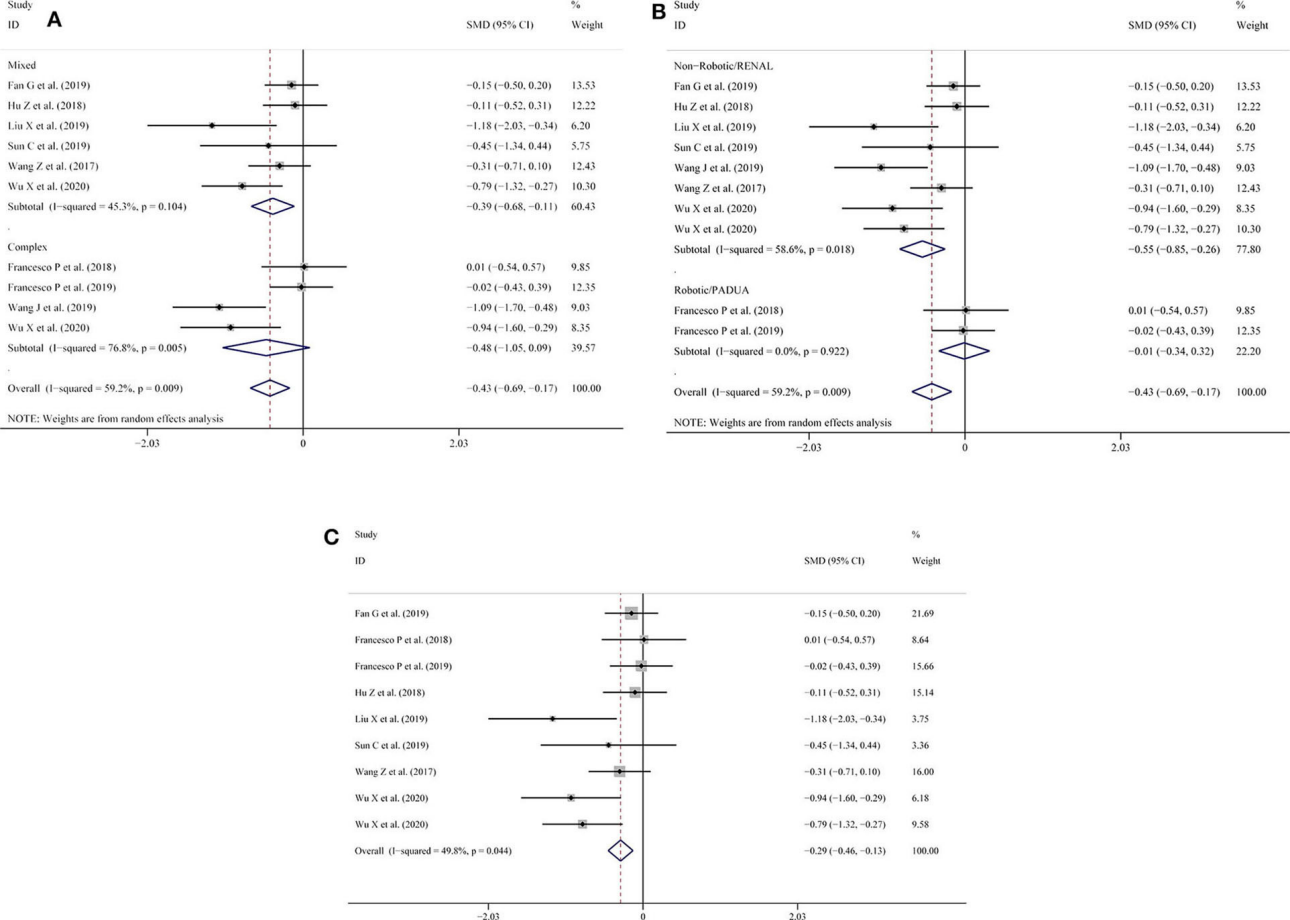
**(A)** Subgroup analysis performed by complexity of the tumor and intraoperative blood loss; **(B)** subgroup analysis performed by type of surgery or nephrometry score for intraoperative blood loss; **(C)** intraoperative blood loss excluding the study by Wang et al.

Similarly, in the intraoperative blood loss subgroup analysis, the results showed that lower heterogeneity was found in the mixed tumor group (SMD = −0.39; 95% CI = −0.68 to −0.11, *I*^2^ = 45.3%, *P* = 0.104; Figure 4A) compared with the complex tumor group (SMD = −0.48; 95% CI = −1.05 to −0.09, *I*^2^ = 76.8%, *P* = 0.005; Figure 4A). When subgroup analysis was performed according to type of surgery or nephrometry score, the heterogeneity was high (SMD = −0.55; 95% CI = −0.85 to −0.26, *I*^2^ = 58.6%, *P* = 0.018; Figure 4B) among the indicated studies with non-robotic surgery or RENAL nephrometry score, but the heterogeneity was low (SMD = −0.01; 95% CI = −0.34 to −0.32, *I*^2^ = 0.0%, *P* = 0.922; Figure 4B) among the indicated studies with robotic surgery or PADUA nephrometry score. To reduce the pooled result of heterogeneity, we consecutively omitted the included studies individually. After Wang et al.'s study (14) was excluded, the heterogeneity of the intraoperative blood loss declined (SMD = −0.29; 95% CI = −0.46 to −0.13, *I*^2^ = 49.8%, *P* = 0.044; Figure 4C).

### Sensitivity Analysis and Publication Bias

**Figure 5:**
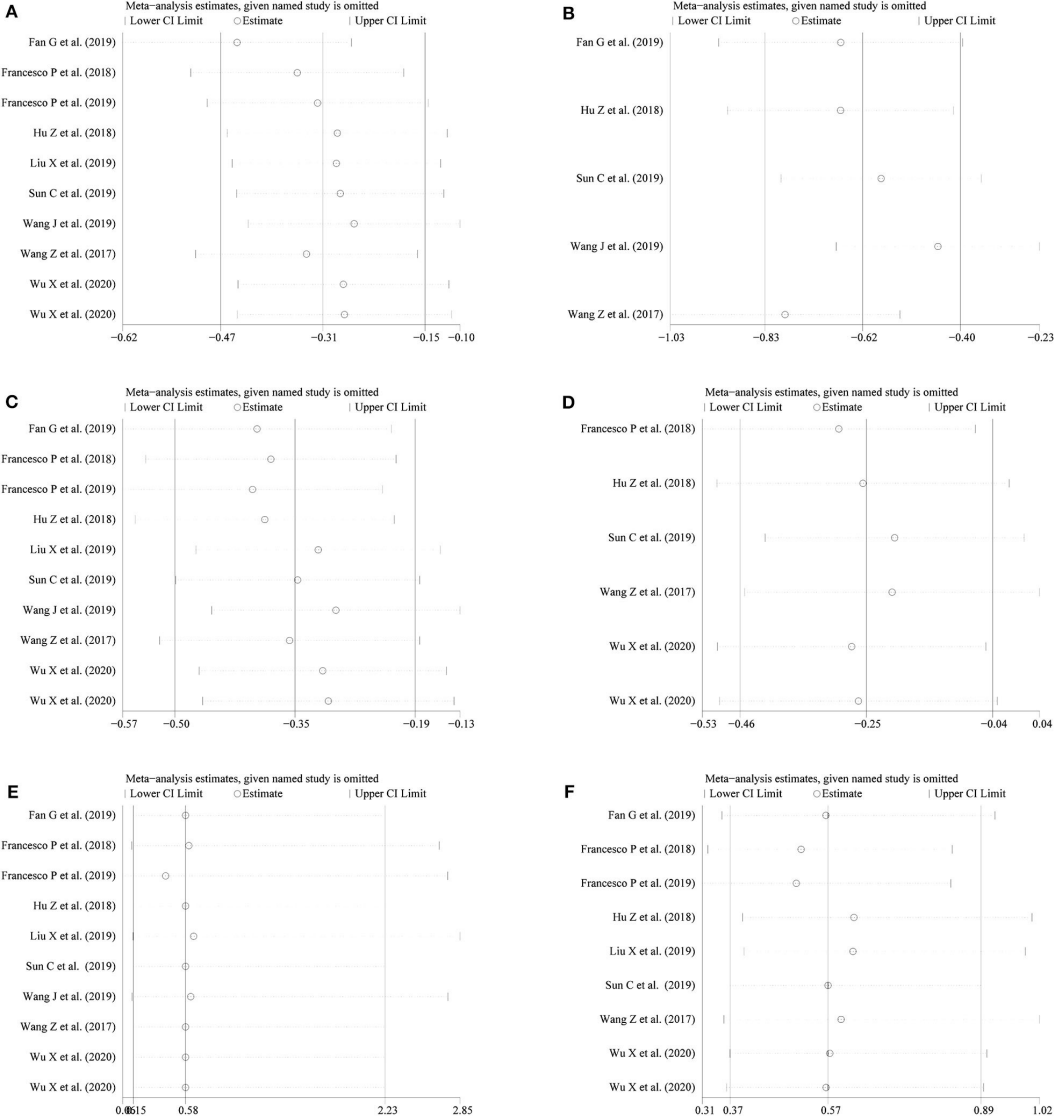
Sensitivity analysis for **(A)** operation time; **(B)** warm ischemia time; **(C)** intraoperative blood loss; **(D)** reduction in eGFR; **(E)** positive surgical margin; **(F)** complications.

**Figure 6:**
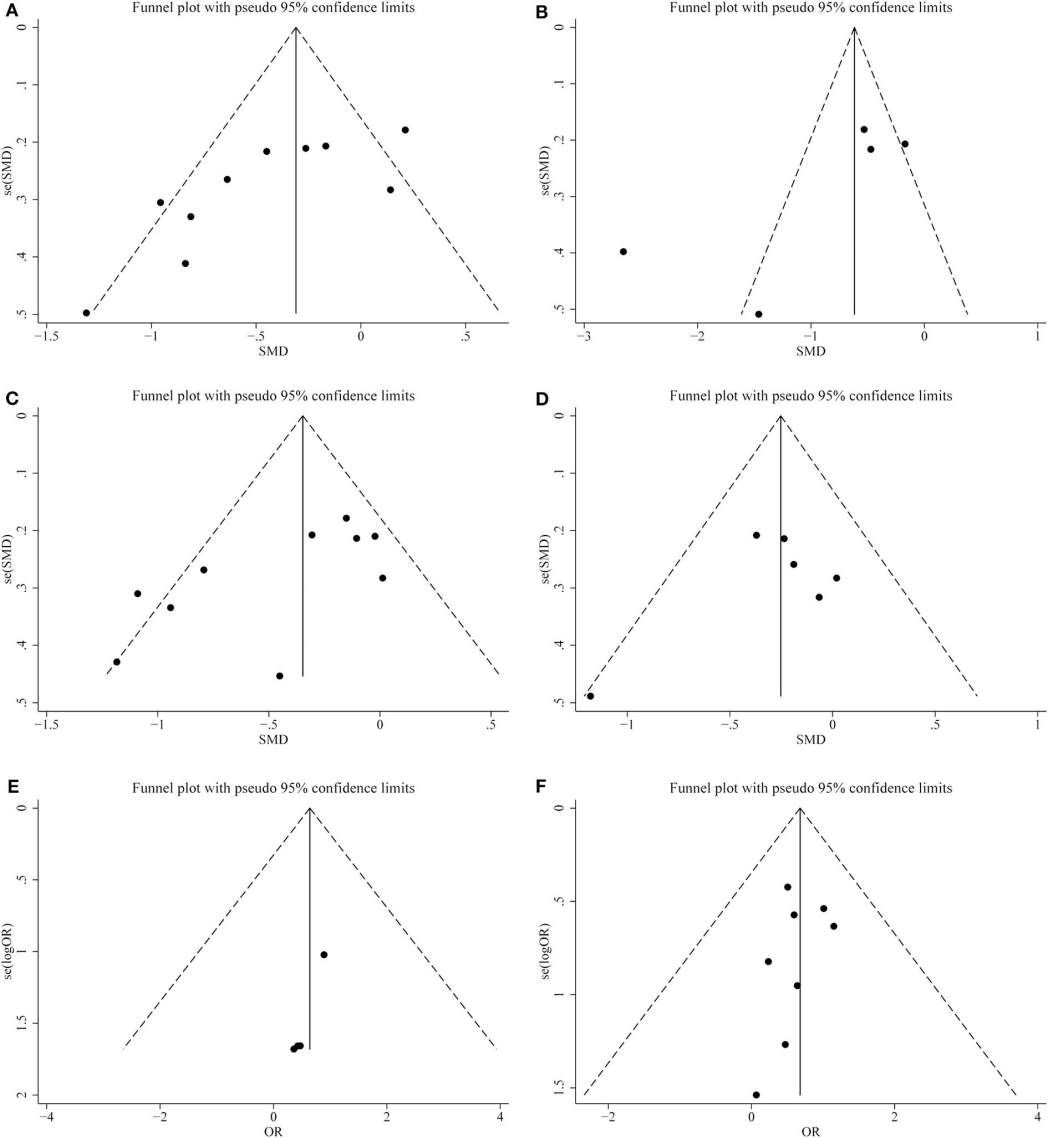
Funnel plots of publication bias for **(A)** operation time; **(B)** warm ischemia time; **(C)** intraoperative blood loss; **(D)** reduction in eGFR; **(E)** positive surgical margin; **(F)** complications.

**Figure 7:**
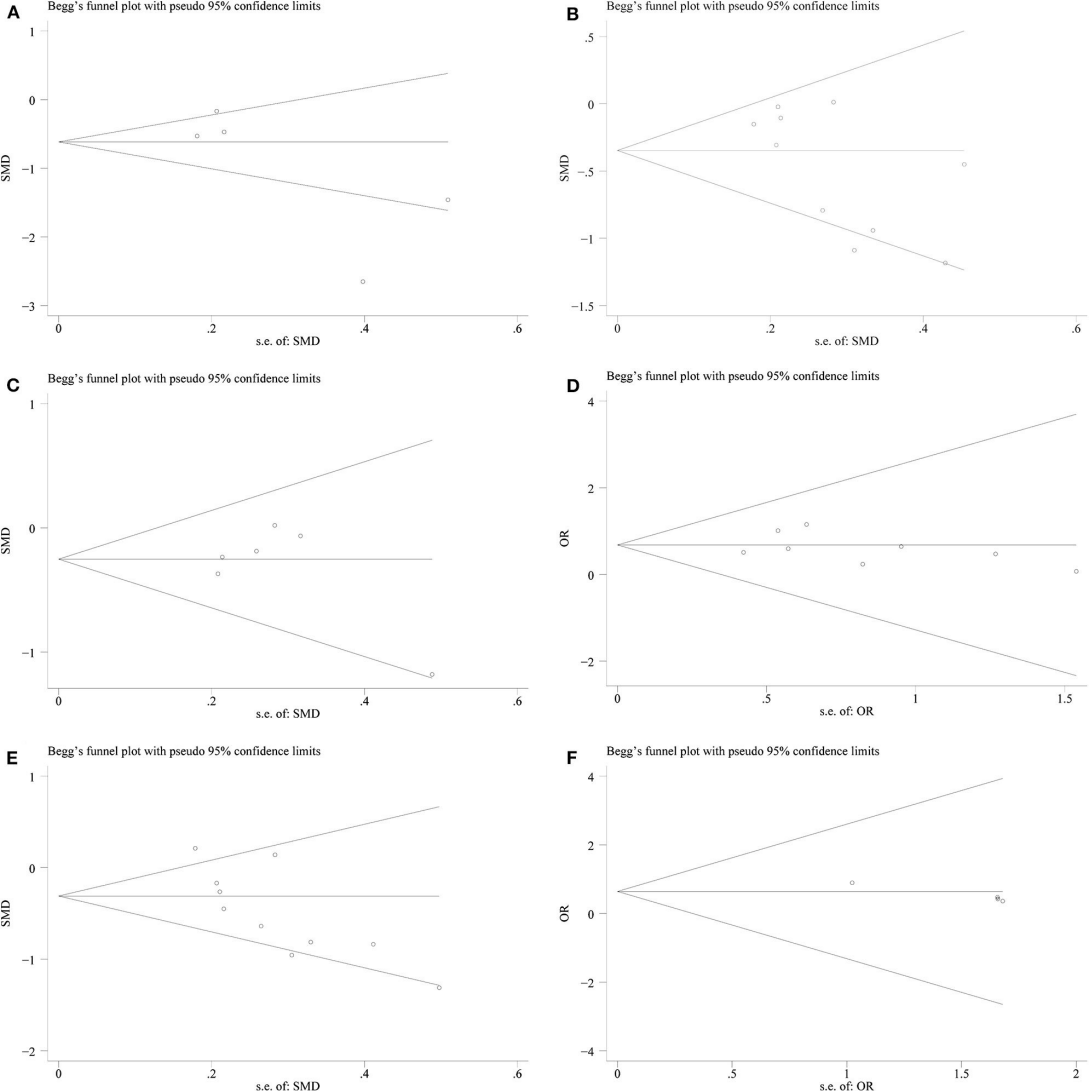
Begg's test plots showing publication bias for **(A)** warm ischemia time; **(B)** intraoperative blood loss; **(C)** reduction in eGFR; **(D)** complications; **(E)** operation time; **(F)** positive surgical margin.

We conducted a sensitivity analysis to examine the effect of a single study on the collective results by consecutively omitting each single study. Due to the different follow-up period of times based on the reduction of eGFR, we could not evaluate its robustness. For other remaining outcomes, except for complications, statistical robustness was evaluated by other methods, as shown in Figure 5. Publication bias was evaluated using Funnel plots and Begg's test. Funnel plots are shown in Figure 6. By using Begg's test, no obvious publication bias was found regarding warm ischemic time (*p* = 0.462, Figure 7A), intraoperative blood loss (*p* = 0.107, Figure 7B), reduction in eGFR (*p* = 0.707, Figure 7C), and complications (*p* = 0.711, Figure 7D). Obvious publication bias was found regarding operation time (*p* = 0.012, Figure 7E) and positive surgical margin (*p* = 0.089, Figure 7F), but further analysis with trim-and-fill test revealed that this publication bias did not impact the initial estimates (no trimming performed; data unchanged).

## Discussion

Renal cell carcinoma is the most common solid lesion in the kidney, which constitutes ~3% of all cancers, with the highest incidence in Western countries (21). Surgery is the only curative treatment for localized renal cell cancer. During PN, in order to obtain clear operative field and precise surgical closure of the collecting system, it is necessary that surgeons clamp the renal pedicle to interrupt renal blood flow during the procedure, especially for renal hilar tumors or those with deep parenchymal invasion. The longer the clamping time of renal pedicle, the greater the impairment of renal function. Warm ischemia time was one of the most important predictors of renal function preservation after LPN. All efforts should be made in order to shorten the warm ischemia time as much as possible, especially when planning to perform LPN for complex renal tumors (22).

Recently, with the advantages of the surgical robotic system, it has been possible for urologists to perform a meticulous microdissection on renal arterial branches feeding the tumor during surgery (23). In addition, several useful scoring systems such as the R.E.N.A.L. (24) and P.A.D.U.A. (25) nephrometry scores have been used to assess the complexity of the tumor.

With rapid development of 3D printing technology in recent years, 3D printing is not only widely applied in industries such as traditional manufacturing and electronics, but it has also gained much interest in the medical field (26). The process of 3D printing involves making a 3D anatomical model via layer by layer printing (27). The procedure of 3D printing in medical practice includes the design of the 3D models based on medical imaging data with computer modeling software, the 3D model is cut into slices, then the model is printed layer by layer (26). Through the 3D anatomical model, surgeons can not only precisely identify location of tumors and direction of the tumor specific arterial branches, as well as quantify the size of the renal defect, but also can predicate the position of the blood vessel and collecting system, which might be damaged during the surgical resection of the tumor (28). With the combination of the abovementioned benefits, 3D printing technology can help surgeons make more meticulous preoperative preparation, as well as make rational choice of operative approach to minimize damage to the surrounding tissue.

In PN, the term Trifecta, indicating negative margin, no complication, and maximal renal function preserve, is used to evaluate the success of a procedure to some extent, which is the ultimate goal for urologists (15). In recent years, an increasing number of urologists have been applying 3D printing technology to preoperative assessment of complex renal tumors. Thus, reports regarding the advantages of 3D-preoperative assessment for the treatment of complex renal tumors have emerged, but these benefits have not been confirmed by evidence-based science. In order to draw a definitive conclusion, we conducted this systematic review and meta-analysis to evaluate the safety and effectiveness of LPN-3DPA.

In our review, we found that patients treated by LPN-3DPA had shorter operation time and warm ischemia time, and less intraoperative blood loss with heterogeneity existed. To facilitate the meta-analysis and to minimize heterogeneity, we excluded the study by Fan et al. (11) in the subgroup analysis for the evaluation of operation time, because this study accounted for the major source of heterogeneity. After we read this article in detail, we identified two explanations for the long operative time. The first was that the operation time reflected the difficulty of the procedure, the technique used by the operator, and surgical experience. The second reason was that for three cases described in the article the surgical modality was switched, which obviously increased the operative time.

As for the analyses of warm ischemia time and intraoperative blood loss, the study by Wang et al. (14) showed high heterogeneity in the sensitivity analyses. Similarly, we excluded it from the analysis of warm ischemia time and intraoperative blood loss. After careful assessment of the study, we concluded that it was a poorly planned retrospective comparative study, which was the reason for the apparent biases. Nonetheless, given the differences in the surgeons' skills, operation conditions, and scope of application of 3D technology, the heterogeneity could not be completely eliminated between studies.

The kidney injury caused by prolonged warm ischemia is an important cause of post-operative acute kidney injury and chronic kidney disease. How to minimize warm ischemic injury in PN and maximize the protection of renal function has always been the focus of national and international experts and scholars. In our meta-analysis, although various studies had different follow-up times for post-operative renal function, the pooled results indicated that the LPN-3DPA group experienced less renal function impairment than the LPN-C2DPA group.

With regard to complications, the incidence of serious complications dropped to about 3 and 3.2% in the LPN and robot-assisted partial nephrectomy groups, respectively (29). In the overall meta-analysis, we found that there were fewer complications in the LPN-3DPA group. Conversely, we did not find any significant differences between the LPN-3DPA and LPN-C2DPA groups in the positive surgical margin, mainly due to the small sample size; besides, it has been reported that the positive margin rate by LPN is very low (only 0.7–4%) (30). Therefore, according to the above meta-analysis, it is clear that the application of a 3D-preoperative assessment not only can speed up the operational procedure but also benefits the prognosis of patients with complex renal tumors.

To the best of our knowledge, this meta-analysis is the first to systematically evaluate the safety and effectiveness of LPN-3DPA in renal tumor patients, and the 10 studies included in the meta-analysis strictly adhered to our inclusion and exclusion criteria with high methodological quality. Therefore, the results of the meta-analysis are generally reliable.

However, there were some limitations in our study. First, only 10 trials met the inclusion criteria after searching various databases, and the included studies were small in sample size. The statistical power to detect the outcomes difference was limited. Further, three studies from the Chinese literature were included, which will not be accessible to non-Chinese researchers. Second, most of the studies included in this meta-analysis were retrospective comparative studies, which were more likely to have been subjected to various biases and high heterogeneity. Third, 3D printing was generally used to assist LPN in patients with complex renal tumors, and the nephrometry score was used to evaluate the complexity of renal tumors. In theory, the higher the nephrometry score, the better the effectiveness of 3D printing assisted LPN. However, due to the lack of original data for each patient in the included literature regarding nephrometry scores, and the small sample size in the studies, we could not address this issue in this analysis. Finally, as small sample size study populations were included in our analysis, we believe that further results from high-quality trials and more rigorous, large-scale, long-term follow-up in RCTs should be provided to update this study.

## Conclusion

Overall, for LPN performed in patients with renal tumors, 3D printing technology can help surgeons obtain more comprehensive information and provide theoretical guidance preoperatively. In our meta-analysis, LPN under the guidance of 3D printing technology is superior to the conventional LPN in terms of operation time, warm ischemia time, intraoperative blood loss, complications, as well as reduction in eGFR. However, the heterogeneity and small sample size in our current study may hamper our meta-analysis, so more RCTs are needed to go a step further in confirming the benefits of combining LPN with 3D printing techniques for the treatment of renal tumors.

## Data Availability Statement

All datasets generated for this study are included in the article/Supplementary Material.

## Author Contributions

YJ, HZ, and JC designed and conceived the research. YJ and HZ searched the database and analyzed the data. YJ, HZ, JC, and ZZ wrote the draft. All authors reviewed the manuscript and approved the final manuscript.

## Conflict of Interest

The authors declare that the research was conducted in the absence of any commercial or financial relationships that could be construed as a potential conflict of interest.

## Abbreviations

LPN: laparoscopic partial nephrectomy
3D: three dimensional
2D: two dimensional
LPN-3DPA: laparoscopic partial nephrectomy with 3D-preoperative assessment
LPN-C2DPA: laparoscopic partial nephrectomy with conventional 2D-preoperative assessment
RCT: randomized controlled trial
PCS: prospective comparative studies
RCS: retrospective comparative studies
Non-RCT: non-randomized controlled trial.
