# Supplementary material for: Three-Dimensional Printing Assisted Laparoscopic Partial Nephrectomy vs. Conventional Nephrectomy in Patients With Complex Renal Tumor: A Systematic Review and Meta-Analysis
Source: Front Oncol. 2020 Oct 22;10:551985. doi: 10.3389/fonc.2020.551985 (PMC7643019; doi:10.3389/fonc.2020.551985)
Supplement: Supplementary file 1 [file Table_1.docx]

Table 1. The characteristics of the included studies

| Study | Year | Study Type | Patients Enrolled | Age(years) | Gender | BMI(kg/m^2^) | Tumor size(cm) | RENAL score | | | PADUA score |
| --- | --- | --- | --- | --- | --- | --- | --- | --- | --- | --- | --- |
|  |  |  | T/C | mean±SD | male/female | mean±SD | mean±SD | Range | T/C | mean±SD | mean±SD |
| Fan G et al. | 2019 | RCS | 69/58 | T:48±11.9 C:50±11.4 | T:38/31 C:26/32 | T:23.7±3 C:23.2±4.4 | T:4±2.6 C:3.9±1.4 | 4-7 8-12 | 33/39 36/19 | NA | NA |
|  |  |  |  |  |  |  |  |  |  |  |  |
| Francesco P et al. | 2018 | PCS | 21/31 | T:60.8±12.3 C:59.5±10.6 | T:15/6 C:23/8 | T:24±1.5 C:25±1.1 | T:5.08±1.61 C:5.09±1.51 | NA | NA | NA | T:11±0.74 C:10.5±0.74 |
|  |  |  |  |  |  |  |  |  |  |  |  |
| Francesco P et al. | 2019 | PCS | 48/43 | T:62±15 C:58±9.8 | T:35/13 C:33/10 | T:24.1±3.7 C:25.9±3.8 | T:4.86±1.87 C:4.46±1.31 | NA | NA | NA | T:11±1.48 C:10±0.74 |
|  |  |  |  |  |  |  |  |  |  |  |  |
| Hu Z et al. | 2018 | RCS | 42/46 | T:50±12.75 C:50.5±14 | T:25/17 C:26/20 | T:24.74±4.5 C:24.6±3.9 | T:3.8±2.0 C:3.6±1.6 | 4-10 | 42/46 | NA | NA |
|  |  |  |  |  |  |  |  |  |  |  |  |
| Liu X et al. | 2019 | RCS | 12/14 | T:53±17.9 C:54±11.3 | T:7/5 C:8/6 | T:22.89±1.9 C:23.1±1.5 | T:3.52±0.97 C:3.96±1.04 | NA | NA | T:6.5±1.9 C:6.3±1.5 | NA |
|  |  |  |  |  |  |  |  |  |  |  |  |
| Sun C et al. | 2019 | RCT | 10/10 | T:50.3±19.2 C:58.1±10.3 | T:7/3 C:9/1 | T:21.8±1.7 C:22.7±1.6 | T:3.2±1 C:3±1 | NA | NA | T:7.20±1.55 C:7.00±1.41 | NA |
|  |  |  |  |  |  |  |  |  |  |  |  |
| Wang J et al. | 2019 | RCS | 21/28 | T:56.25±5.75 C:60±6 | T:15/6 C:17/11 | T:23.25±2.25 C:24±1.75 | T:3.2±0.55 C:3.3±0.475 | 8-12 | 21/28 | T:10±1 C:10±0.75 | NA |
|  |  |  |  |  |  |  |  |  |  |  |  |
| Wang Z et al. | 2017 | RCS | 49/45 | T:53.9±8.6 C:56.8±7.6 | T:29/20 C:22/23 | T:22.1±1.6 C:22.0±1.8 | T:3.2±1.5 C:3.4±1.6 | 4-7 8-12 | 27/27 22/18 | T:7.3±1.7 C:6.9±1.8 | NA |
|  |  |  |  |  |  |  |  |  |  |  |  |
| Wu X et al. | 2020 | RCS | 20/20 | T:58.95±11.69 C:54.15±11.90 | T:15/5 C:14/6 | T:25.12±2.75 C:24.98±2.61 | T:5.05±0.63 C:4.95±0.67 | 4-6 7-9 10-12 | 3/3 14/15 3/2 | NA | NA |
|  |  |  |  |  |  |  |  |  |  |  |  |
| Wu X et al. | 2020 | RCS | 30/30 | T:57.6±11.7 C:56.4±9.8 | T:22/8 C:21/9 | T:25.2±2.8 C:24.9±2.5 | T:4.0 C:3.75 | 4-6 7-9 10-12 | 11/12 17/15 2/3 | NA | NA |
|  |  |  |  |  |  |  |  |  |  |  |  |

T:3D group；C: Conventional group; SD: standard deviation; RCS: retrospective comparative studies; RCT: randomized controlled trial; PCS: prospective comparative studies; BMI: body mass index; NA: Not available.
